# Unveiling prognostic genes and regulatory mechanisms of stress granules in gastric cancers: an integrated analysis of bulk transcriptomics and single-cell RNA sequencing

**DOI:** 10.3389/fonc.2026.1750088

**Published:** 2026-02-18

**Authors:** Ruilong Kou, Chenyu Zhu, Yu Chen, Jinzhou Wang, Jiuhua Xu, Bin Lan, Zhiwei Qin

**Affiliations:** 1 Department of Gastrointestinal Surgery 2 Section, The First Affiliated Hospital, Fujian Medical University, Fuzhou, China; 2 Department of Gastrointestinal Surgery, National Regional Medical Center, Binhai Campus of the First Affiliated Hospital, Fujian Medical University, Fuzhou, China; 3 Department of Gastrointestinal Surgery, Affiliated Hospital of Guilin Medical University, Guilin, China; 4 First Affiliated Hospital of Dalian Medical University, Dalian, China

**Keywords:** bulk transcriptomics, gastric cancer, risk model, single-cell analysis, stress granules

## Abstract

**Background:**

Gastric cancer (GC) is often associated with a poor prognosis, and the precise molecular mechanisms driving its pathogenesis are not yet fully characterized. Stress granules (SGs) are now understood to play a crucial role in tumor progression, yet the prognostic value of SG-related markers in GC remains unclear. This study aimed to identify SG-related prognostic genes, clarify their clinical and biological significance in GC, and validate their potential as predictive indicators for patient overall survival (OS).

**Methods:**

Single-cell and transcriptomic data for gastric cancer, along with genes related to stress granules (SGRGs), were acquired from public databases and literature. Candidate genes were identified by intersecting differentially expressed genes (DEGs) with SGRGs. Prognostic genes were identified through univariate Cox regression, and a risk score model was constructed. The model’s performance was validated in an independent cohort. Based on risk stratification, functional enrichment analysis, immune cell infiltration pattern assessment, and chemotherapy drug sensitivity analysis were conducted. Cell types expressing the prognostic genes were identified using single-cell RNA sequencing (scRNA-seq), and the related key cell clusters were identified.

**Results:**

*SERPINE1*, *CD36*, *MMRN1*, and *GRP* were identified as prognostic genes. The risk model demonstrated good performance in predicting the survival status of GC patients. GSEA revealed that significantly enriched pathways included neuroactive ligand-receptor interaction and extracellular matrix (ECM)-receptor interaction pathways. CD36, MMRN1, and SERPINE1 demonstrated significant positive correlations with mast cells (correlation coefficients (r) > 0.3, *P* < 0.001). Chemotherapy drugs exhibited greater efficacy in high-risk GC patients. Moreover, endothelial cells were considered key cells and played a critical role in GC. Finally, SERPINE1 expression was associated with clinical features and prognosis in GC.

**Conclusion:**

In summary, we identified a four-gene SG-related signature strongly associated with prognosis in GC and constructed a predictive model with clinical potential. Our integrated analysis identified endothelial cells as a candidate population linked to the expression of these genes. These findings provide associative evidence linking SGs to GC outcomes and highlight potential targets for future mechanistic and therapeutic exploration.

## 1 Introduction

Gastric cancer (GC) stands as a major burden in modern cancer medicine, currently positioned as the world’s fifth most frequently diagnosed malignancy while simultaneously serving as the fourth predominant contributor to cancer mortality. According to 2022 global cancer statistics, the worldwide incidence of malignancy reached 19.96 million, among which gastric cancer cases accounted for 968,400, representing 4.9% of the total global cancer burden (1). Although its incidence has declined in some regions, it remains a major health issue in high-prevalence areas such as East Asia (2).

GC is a multifactorial malignancy resulting from the interplay of environmental and genetic factors, including Epstein-Barr virus (EBV) infection, smoking, alcohol consumption, family history, and diet (3, 4). The insidious nature of GC, characterized by limited precancerous symptoms and early manifestations, results in most patients being diagnosed at advanced stages, thereby missing the window for curative surgical resection (5). Although the implementation of endoscopic screening has reduced GC mortality by 30% and achieved 5-year survival rates of 60-80% in patients with stage IA and IB tumors, the overall clinical prognosis remains challenging (6). The median overall survival for advanced GC remains approximately 8 months despite standard treatments that combine neoadjuvant chemoradiotherapy, molecular-targeted therapy, and immunotherapy (7). The marked inter-patient heterogeneity in clinical outcomes, compounded by postoperative recurrence and limited therapeutic efficacy, underscores the urgent need to identify novel prognostic biomarkers and develop optimized predictive models for personalized GC management with direct clinical translation potential.

Stress granules (SGs) are non-membranous ribonucleoprotein complexes assembled from mRNA, RNA-binding proteins, and translation initiation factors in response to various stress stimuli (8, 9). These dynamic structures play pivotal roles in tumor initiation and progression, with stress adaptation becoming an important hallmark of cancer cells (10–12). Compared with normal cells, SG-related components are upregulated in various tumor cells, including G3BP1 and G3BP2, and elevated SG expression has been correlated with poor prognosis in cancer patients (13, 14). Growing evidence demonstrates that cancer cells generate SGs to protect critical mRNAs from degradation, thereby promoting survival, metastasis, and therapeutic resistance to chemotherapy and radiotherapy (15, 16). Using GC as a specific example, the long non-coding RNA TM4SF1-AS1 interacts with a range of SG-associated proteins, namely G3BP2, RACK1, and DDX3. Through sequestration of RACK1 into SG compartments, TM4SF1-AS1 facilitates SG assembly while suppressing apoptotic processes in GC cellular populations (17). Besides, ubiquitin-associated protein 2-like (UBAP2L) mediates oxaliplatin resistance by orchestrating the formation of nuclear SGs. This process involves RACK1 recruitment and AKT-mediated HSF1 activation, ultimately enhancing the transcriptional upregulation of UBAP2L (18). However, the specific functions of stress granule-related genes (SGRGs) in gastric cancer prognosis remain unclear. Addressing this knowledge gap is crucial for exploring potential therapeutic targets.

To address this challenge, single-cell RNA sequencing (scRNA-seq) has emerged as a transformative technology in transcriptomics, enabling comprehensive cellular profiling to characterize complex biological processes at high resolution (19). In contrast to conventional bulk sequencing approaches, single-cell RNA sequencing (scRNA-seq) elucidates intercellular heterogeneity, identifies rare cellular subpopulations, and enables a high-resolution characterization of cellular dynamics across diverse physiological and pathological states (20). This powerful approach has become instrumental in dissecting the heterogeneity and complexity of RNA transcripts within individual cells while revealing the complex composition of different cell types and functions within biological organisms (21). For example, through single-cell analysis of alpha-fetoprotein-positive gastric cancer, a malignant cell subset with unique copy number variation characteristics was identified, revealing the molecular regulatory networks associated with its abnormal proliferation and metastasis (22). The integration of bulk transcriptomics with single-cell analysis provides valuable insights into disease mechanisms and cellular interactions that drive pathological processes.

This study aimed to systematically investigate the correlation between SGs and GC prognosis using comprehensive transcriptomic and single-cell data from public databases. Through integrated bioinformatic analysis, we established and validated an SGRG-based prognostic signature, constructed robust predictive models for clinical application, and delineated the distinct biological processes and immune infiltration patterns across risk cohorts. Our investigation also included drug-sensitivity profiling and single-cell-resolution identification of key cell types driving GC progression. Taken together, these findings provide novel insights into SG-mediated mechanisms in GC pathogenesis while offering valuable translational tools for clinical prognosis prediction and potential immunotherapeutic strategies, potentially advancing personalized treatment approaches for this patient population.

## 2 Materials and methods

### 2.1 Data collection

Transcriptomic profiling via RNA-seq, survival records, and genomic somatic mutation profiles, along with clinicopathological parameters of patients diagnosed with GC, were retrieved from The Cancer Genome Atlas (TCGA) database (https://portal.gdc.cancer.gov/), with data acquisition completed on March 7, 2025. The GC dataset (TCGA-STAD) contained 36 normal tissue samples and 412 GC tissue samples. Of these 412 GC samples, survival information was available for 385 corresponding patients. This dataset was utilized as a training set. From the Gene Expression Omnibus (GEO) database (https://www.ncbi.nlm.nih.gov/geo/), a cohort comprising 357 GC tissue specimens was extracted from dataset GSE84433, which was generated using the GPL6947 platform. The 357 GC samples, which included survival information and gene expression data, were available for validation. Single-cell RNA sequencing data archived as GSE167297 (generated on platform GPL20301) encompassed 5 specimens derived from GC tissues alongside 4 specimens from normal gastric tissues. In addition, a total of 844 SGRGs were included in this study. This gene set was derived from a previously published study (23) and specifically obtained by searching the GeneCards database (https://www.genecards.org) using the keyword “Stress granules”, with the screening criterion that the gene relevance score was greater than 4. Detailed information regarding these genes is provided in **Supplementary Table S1**.

### 2.2 Differential expression analysis

To screen DEGs in the training cohort, differential expression profiling comparing malignant GC specimens with normal gastric tissue controls was performed using the DESeq2 package (v3.54.0), with |log_2_Fold Change (FC)| > 1.5 and *P* < 0.05 (24). A volcano plot was generated to visualize the distribution of DEGs through the implementation of the ggplot2 package (v3.5.1). Within this graphical representation, the 10 most significantly altered genes—ranked according to descending |log_2_FC| values and exhibiting either marked upregulation or downregulation—were specifically annotated. Subsequently, a hierarchical clustering heatmap illustrating the expression patterns of these significantly altered genes was generated using the pheatmap package (v0.7.7) (25, 26).

### 2.3 Identification, functional enrichment, and protein-protein interaction network of candidate genes

To obtain the genes related to SGs that were differentially expressed in GC, the ggvenn package (v0.1.10) was applied to obtain the intersection of DEGs and SGRGs. The resulting overlapping genes were defined as candidate genes (27). To elucidate the biological pathways and functional characteristics associated with the candidate gene set, enrichment analysis was performed using the clusterProfiler package (v 4.10.1). This analytical approach encompassed GO annotation across three ontological domains, molecular function (MF), biological process (BP), and cellular component (CC), alongside KEGG pathway mapping, with statistical significance defined as P < 0.05 (28). The candidate gene list was submitted to the STRING database (https://string-db.org/) to generate PPI networks, applying a confidence score cutoff of 0.4 to delineate protein-level interactome relationships among the selected genes. A graphical representation of these molecular interactions was subsequently generated using Cytoscape software (version 3.10.2) (29).

### 2.4 Identification of prognostic genes

To identify genes with significant prognostic relevance for OS in GC patients, univariate Cox proportional hazards regression was performed using the survival package (v3.7-0) on 385 GC cases with complete survival data. Selection criteria mandated HR values deviating from unity coupled with *P* < 0.001 (30). Subsequently, the PH assumption was validated for these candidate prognostic markers. Genes with a *P*-value greater than 0.05 were retained for subsequent analysis, while those with a *P*-value below this threshold were excluded. Genes that satisfied the PH assumption criteria were designated as validated prognostic genes for downstream analyses.

### 2.5 Construction and validation of a risk model

Using the 385 GC specimens with accompanying survival metrics from the training cohort, we first performed missing data preprocessing—removing samples with missing clinical indicators and genes with missing expression values—and then constructed an RSF-based predictive framework via the randomForestSRC package (v 3.2.3). The core parameters were strictly standardized to ensure reproducibility: the number of trees (ntree) was set to 14, the number of variables sampled for each split (mtry) was fixed at 1, and a consistent random seed (set.seed = 1) was applied throughout the model training and prediction processes. Nodes were split using the log-rank test to maximize survival difference, tailoring the model for OS prediction. This machine learning method ranked prognostic genes using the variable importance score (VIMP), which quantifies the reduction in model prediction accuracy when a specific gene is excluded, and provided personalized risk scores for each sample. RSF analysis was performed to confirm the importance of the pre-screened genes rather than to further select variables; by calculating the median risk score in the training cohort, 385 gastric cancer patients were divided into the high-risk group (HRG) and the low-risk group (LRG). Next, Kaplan-Meier (K-M) survival curves were generated using the survival package (v3.7-0), and the OS differences between the two groups were analyzed through the log-rank test (*P* < 0.05). Next, time-dependent receiver operating characteristic (ROC) curve analysis was performed using the survivalROC package (v1.0.3.1) to evaluate discriminative ability at 3, 5, and 7 years (AUC > 0.6 was the benchmark for acceptable performance) (31). A comprehensive validation procedure was adopted in the independent GSE84433 cohort to determine the prediction accuracy and model stability, thereby confirming the generalizability of the risk stratification framework.

### 2.6 Distribution of the risk score in the clinical characteristics

The clinical utility of the constructed risk stratification model in GC was appraised by examining the risk score distribution across 385 patients in the training cohort, incorporating comprehensive clinicopathological variables, including age, gender, overall stage, T stage, N stage, and M stage. Notably, the Wilcoxon test was used to compare differences in risk scores among subgroups stratified by age (age > 67, age ≤ 67), gender (male, female), M stage (M0, M1), stage (Stage I, Stage II, ≥ Stage III), T stage (T1, T2, T3, T4), and N stage (N0, N1, N2, N3).

### 2.7 Gene set enrichment analysis and immune microenvironment analysis

To explore pathway-level biological characteristics that distinguish HRG from LRG in GC patients, GSEA was performed. The annotated gene collection c2.KEGG.v2022.1.Hs.symbols, retrieved from MSigDB (https://www.gsea-msigdb.org/gsea/msigdb), served as the reference repository for this enrichment analysis. Differential expression profiling comparing HRG versus LRG was performed using the DESeq2 package (v 3.54.0) across the 385 GC specimens with survival metrics. Following this analysis, genes were hierarchically ordered by descending log2 FC. GSEA was conducted utilizing the clusterProfiler package (v4.10.1), with thresholds of |normalized enrichment score (NES)| > 1 and *P* < 0.05.

The immune microenvironment plays a crucial role in tumorigenesis. According to the single-sample gene set enrichment analysis (ssGSEA) algorithm, the GSVA package (v 1.50.0) was used to calculate the infiltration scores of 28 immune cell types between HRG and LRG in 385 GC samples with survival information (32, 33). Subsequently, the Wilcoxon rank-sum test was conducted to evaluate differences in immune cell infiltration landscapes between the two risk-stratified cohorts, with a statistical significance threshold set at *P* < 0.05.

### 2.8 Gene mutation and chemotherapeutic drug sensitivity analyses

Somatic mutation profiles characterizing HRG and LRG were obtained by extracting relevant genomic alteration data from TCGA. Leveraging somatic alteration information derived from the training cohort, the maftools package (v2.18.0) was deployed to identify and characterize the 20 most frequently mutated genes within HRG and LRG, with emphasis on their association with TMB (34). Comparative analysis of TMB metrics between these dichotomized risk categories was performed using Wilcoxon rank-sum testing, with a significance cutoff of *P* < 0.05.

To investigate the therapeutic effects of chemotherapeutic drugs in GC patients, we obtained 138 chemotherapeutic agents from the Genomics of Drug Sensitivity in Cancer (GDSC) database (https://www.cancerrxgene.org/). Pharmacological sensitivity profiles, quantified as IC_50_ values for individual chemotherapeutic agents, were computed using the pRRophetic package (v0.5) from the 385 GC specimens in the training cohort (35). A comparative evaluation of IC_50_ metrics between HRG and LRG was conducted using Wilcoxon rank-sum testing, with a significance threshold set at *P* < 0.05. For each risk-stratified cohort, the top five statistically significant therapeutic agents were presented. Furthermore, associative relationships associating risk scores to therapeutically significant agents were examined using the psych package (v2.4.3), applying stringent criteria requiring absolute correlation coefficients exceeding 0.3 and *P* < 0.05.

### 2.9 Localization, functional enrichment, and regulatory network analyses of prognostic genes

The location of genes on chromosomes is critical for gene expression and regulation. In this study, the RCircos package (v 1.2.2) was used to determine the chromosomal localization of prognostic genes (36). To further elucidate the mechanisms of action of prognostic genes, the subcellular localizations of these genes were predicted using the mRNALocater database (http://bio-bigdata.cn/mRNALocater/).

To comprehensively elucidate the molecular interplay and functional associations among the prognostic gene signatures, a co-expression network framework was assembled using the GeneMANIA platform (http://www.genemania.org/). GSEA was implemented to decode pathway-level roles of prognostic markers throughout GC pathogenesis. The hallmark gene collection h.all.v2024.1.Hs.symbols.gmt, sourced from MSigDB, constituted the reference annotation resource. Spearman’s rank correlation coefficients were computed between individual prognostic markers and the entire transcriptomic repertoire across training cohort specimens, leveraging the psych package (v2.4.3). Following coefficient derivation, genes were hierarchically ordered by descending Spearman correlation coefficients relative to each prognostic marker, thereby generating the ranked gene list used as input to subsequent GSEA procedures. Enrichment analysis was then performed using the clusterProfiler package (v 4.10.1), employing stringent selection criteria that required absolute normalized enrichment scores exceeding unity and statistical significance at *P* < 0.05.

To comprehensively delineate upstream regulatory mechanisms governing prognostic gene expression, cognate miRNAs targeting these markers were computationally predicted using the miRDB database (https://mirdb.org/). Besides, TFs potentially orchestrating the transcription of prognostic genes were identified using the JASPAR repository (http://jaspar.genereg.net/) integrated into the NetworkAnalyst platform (https://www.networkanalyst.ca/). Graphical representations of both miRNA-mRNA and TF-mRNA regulatory circuits were subsequently rendered via Cytoscape software (v3.10.2).

To identify drugs that might interact with prognostic genes, potential drug candidates targeting these genes were analyzed using the Drug-Gene Interaction database (DGIdb, https://dgidb.org/) . The results were analyzed using Cytoscape (v3.10.2) to visualize the gene-drug interaction network.

### 2.10 Treatment of scRNA-seq data and selection of key cells

Quality assurance procedures for the GSE167297 dataset were implemented using the Seurat package (v5.1.0), applying stringent filtering thresholds: nFeature_RNA between 200 and 5,000, nCount_RNA between 200 and 25,000, and a mitochondrial transcript percentage below 25% (37). Following quality filtration, the FindVariableFeatures function facilitated identification of 2,000 HVGs exhibiting maximal transcriptional variability for downstream analytical pipelines, with the top 10 genes demonstrating the greatest variance being specifically annotated. Normalization of expression matrices was performed using the ScaleData function in the Seurat package (v5.1.0). Principal component analysis (PCA) was conducted on the top 2,000 HVGs. Subsequently, from the results obtained via the JackStrawPlot function, the top 50 principal components (PCs) were selected (*P* < 0.05). To integrate cells from different samples and remove batch effects, the Harmony algorithm was applied. Harmony was applied to the PCA space (using the top 30 PCs) with the sample origin as the batch covariate. This method iteratively aligned cells from different batches in a shared low-dimensional space, effectively mitigating technical variability while preserving biological heterogeneity. Subsequently, with a resolution parameter established at 0.3, unbiased clustering procedures were executed across the entire cellular population, leveraging the synergistic application of FindNeighbors and FindClusters functions. A graphical representation of the clustering patterns was then obtained using the UMAP algorithm, which facilitates dimensionality reduction while preserving topological structure. Cell types of the different clusters were identified after dimensionality reduction and clustering, as previously described (38). Cell type annotation outcomes were mapped onto UMAP coordinate space, with expression profiles of lineage-defining marker genes delineated across distinct cellular populations. Bar chart representations were employed to quantify the relative abundance of individual cell types throughout the entire sample collection. To elucidate cellular functional characteristics at the molecular pathway level, enrichment analysis was conducted utilizing the pathways function integrated within the ReactomeGSA package (v1.16.1) (39).

To analyze the expression patterns of prognostic genes, bubble plots were generated to visualize gene expression across different cell populations. Subsequently, the Wilcoxon test was applied to compare the expression differences of prognostic genes between GC samples and normal samples across various cell types (*P* < 0.05). Cells exhibiting the highest differential expression of prognostic genes were designated as key cells. To further validate and characterize the molecular characteristics of the identified key cells, we analyzed the expression of classic endothelial cell markers at the single cell level (GSE167297). Using the DotPlot function in the Seurat package (v5.1.0), we visualized the expression distributions of the marker genes VEGFA, VWF, and CDH5 across different cell types. Besides, using the ggplot2 package (v3.5.1), we compared the expression differences of these markers between GC samples and normal samples to reveal their specific changes in the gastric cancer microenvironment.

### 2.11 Cell communication, pseudotime, and malignant cell analyses

To investigate the communication network among annotated cells, the CellChat package (v1.6.1) was used to analyze the communication relationships in the GSE167297 dataset and to establish a communication network (40). Subsequently, intercellular communication networks were decoded using the implementation of the CellChat package (v1.6.1), which systematically predicted L-R pairing interactions among annotated cellular subsets. Concurrently, to trace developmental trajectories of pivotal cellular populations and monitor prognostic gene expression dynamics across sequential differentiation states, UMAP-based dimensionality reduction coupled with clustering procedures was applied to these key cell subsets, with a resolution parameter maintained at 0.3. Trajectory inference was subsequently performed using the Monocle package (v2.30.1), which reconstructed the pseudotemporal ordering of cellular states (41). Concurrently, temporal expression dynamics of prognostic markers were profiled along the inferred pseudotime continuum, revealing transcriptional alterations accompanying cellular progression.

Given that cells exhibit multiple normal and malignant states, the diversity among cancer cells is closely associated with GC-specific carcinogenic drivers. To elucidate the heterogeneity of key cells, data from key cells and reference cells were extracted. The InferCNV package (v1.22.0) was then employed to assess copy number variations (CNVs) in key cells, using reference cells as a background to distinguish malignant from non-malignant cells (22). Furthermore, the relative CNV scores of each key cell subpopulation were measured against those of the reference cells.

### 2.12 Clinical analysis based on SERPINE1 expression

Within the training cohort comprising 385 GC specimens harboring survival metrics, patients were dichotomized into high- and low-SERPINE1 expression groups based on the median expression level. The K-M survival curves were generated utilizing the survival package (v3.7-0) to investigate the differences in OS rates between 2 groups, employing the log-rank test with a significance threshold of *P* < 0.05. Besides, the survivalROC package (v 1.0.3.1) was utilized to generate ROC curves (with an AUC > 0.6) corresponding to the 3-, 5-, and 7-year time intervals. Among the 385 GC samples with clinical characteristics in the training set, the samples were divided into 2 groups based on the median SERPINE1 expression level: a high-expression group and a low-expression group. To assess potential associations between SERPINE1 transcriptional levels and diverse clinicopathological parameters, Wilcoxon rank-sum tests were used to evaluate heterogeneity in SERPINE1 expression across distinct clinical feature categories. Besides, a comprehensive comparative analysis of clinicopathological attributes between these two expression-stratified cohorts was conducted. Chi-square testing was applied to categorical parameters, while Student’s t-test was used for continuous variables, with a statistical significance threshold set at *P* < 0.05.

### 2.13 qRT-PCR

Experimental validation of expression profiles for four candidate genes, *SERPINE1*, *CD36*, *MMRN1*, and *GRP*, derived from computational bioinformatics screening, was performed using qRT-PCR across 44 matched pairs of primary GC specimens and their corresponding adjacent non-neoplastic gastric tissues. Global RNA isolation was performed using the RNAiso Plus reagent (Takara, Zihe, Japan) according to the supplier’s protocol. Subsequent quantification of RNA concentration and assessment of RNA purity were performed using a microvolume spectrophotometer (BIO-DL, Shanghai, China). Reverse transcription to generate first-strand cDNA was performed using the PrimeScript™ RT reagent Kit, with 600 ng of purified total RNA as the template.

Amplification reactions for qRT-PCR were performed leveraging PerfectStart Universal Green qPCRSuperMix (Quantobio, Beijing, China) within a ViiA7 Real-Time PCR System (Applied Biosystems). Thethermal protocol encompassed an initial denaturation phase at 95°C lasting 1 minute, followed by 40 iterative cycles consisting of denaturation at 95°C for 5 seconds coupled with annealing/extension at 60°C for 30 seconds. GAPDH served as the internal reference gene. All reactions were performed in triplicate to ensure technical reproducibility. The primers used for the experiments are listed in **Supplementary Table S8**. Relative mRNA levels were calculated using the 2^−ΔΔCt^ method.

### 2.14 Statistical analysis

Comprehensive statistical computations were executed via the R software platform (v4.3.2). For binary group comparisons, Wilcoxon rank-sum testing was implemented with a significance threshold at *P* < 0.05. Nominal categorical data underwent chi-square analysis, whereas numerical continuous measurements were subjected to Student’s t-test, maintaining *P* < 0.05 as the statistical significance cutoff. Notably, in our study, **** represented *P* < 0.0001, *** represented *P* < 0.001, ** represented *P* < 0.01, * represented *P* < 0.05, and ns represented *P* > 0.05.

## 3 Results

### 3.1 Identification of candidate genes associated with SGs in GC

**Figure 1:**
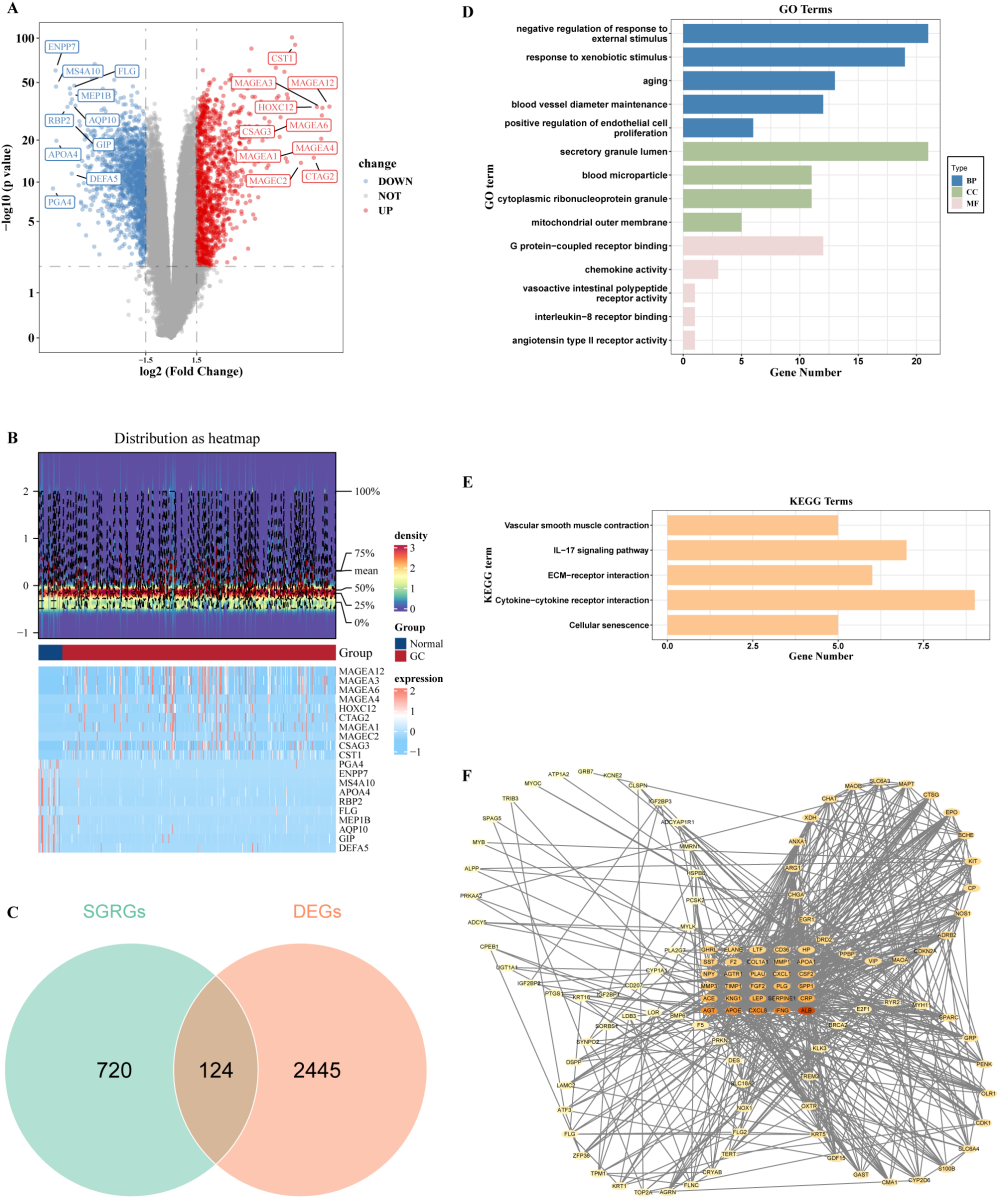
Identification and functional exploration of candidate genes. **(A)** Volcano plot of DEGs in GC. **(B)** Heatmap of DEGs in GC. **(C)** Venn diagram showing 124 differentially expressed SGRGs. **(D)** Bar chart revealing the outcomes of GO functional enrichment of 124 differentially expressed SGRGs. **(E)** Bar chart revealing the outcomes of KEGG pathways enrichment of 124 differentially expressed SGRGs. **(F)** PPI network.

Comparative transcriptomic analysis of malignant GC specimens against normal gastric tissue controls revealed 2,569 significant DEGs, with 1,218 upregulated and 1,351 downregulated in the GC cohort (**Figures 1A, B**). Furthermore, the intersection of DEGs and SGRGs yielded 124 genes, which were subsequently classified as candidate markers for downstream investigation (**Figure 1C**). Functional enrichment analysis of the 124 candidate genes revealed associations with 2,526 GO terms, including 2,195 BPs, 207 MFs, and 124 CCs (*P* < 0.05) (**Supplementary Table S2**). These terms encompassed a wide range of functions such as negative regulation of response to external stimulus, blood vessel diameter maintenance, and angiotensin type II receptor activity (**Figure 1D**). Besides, KEGG pathway analysis demonstrated significant enrichment in 70 pathways, including the IL-17 signaling pathway and ECM-receptor interaction (**Figure 1E**, **Supplementary Table S3**). Subsequently, an interactome network architecture encompassing 872 protein-level interaction edges spanning 119 candidate genes was assembled, thereby delineating the molecular connectivity landscape (**Figure 1F**). In this network, ALB, IFNG, CXCL8, and APOE exhibited frequent protein-level interactions with other genes.

### 3.2 A risk model based on 4 prognostic genes demonstrated superior predictive power in GC

**Figure 2:**
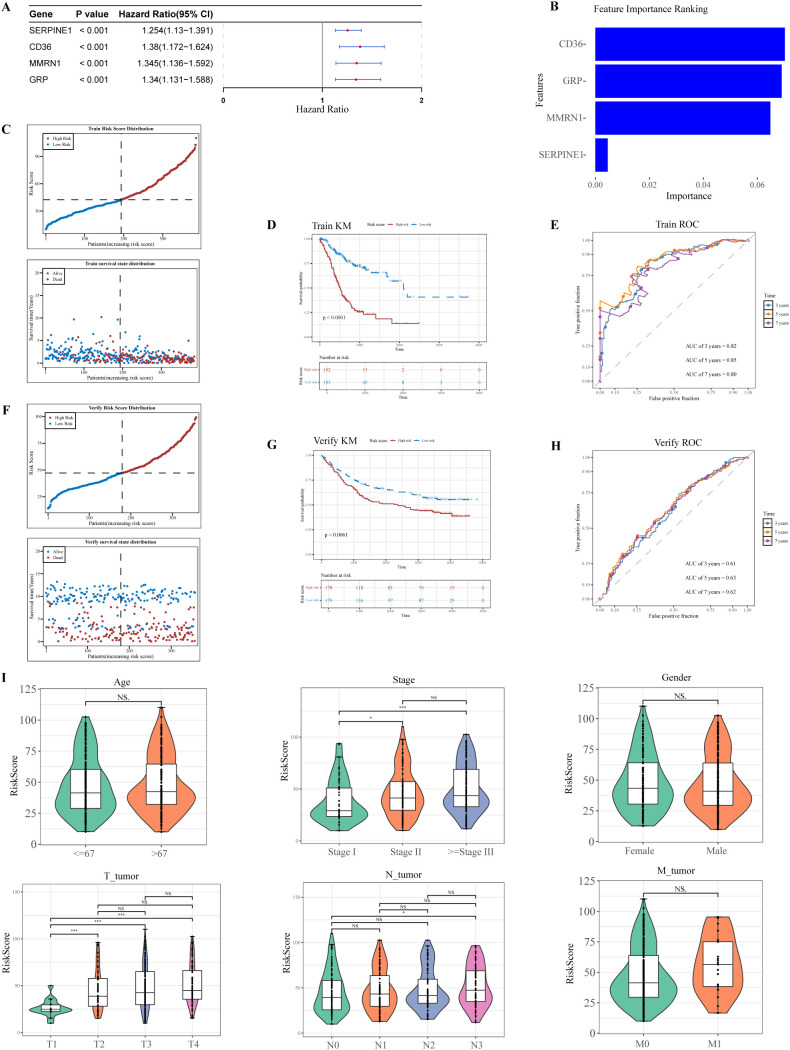
Construction and verification of the risk model. **(A)** Forest plot of univariate Cox regression analysis screening 4 prognostic genes linked to survival. **(B)** Random Forest analysis. **(C)** Distribution of survival status and risk score in the TCGA cohort. **(D)** K-M curves illustrating significant differences in patient survival between risk subgroups in the TCGA cohort. **(E)** ROC curves evaluating the predictive accuracy of the risk model in the TCGA cohort. **(F)** Distribution of survival status and risk score in the GEO cohort. **(G)** K-M curves illustrating significant differences in patient survival between risk subgroups in the GEO cohort. **(H)** ROC curves evaluating the predictive accuracy of MIRGs in the GEO cohort. **(I)** Clinicopathologic characteristics of TCGA in the risk-score. *P < 0.05, **P < 0.01, ***P < 0.001.

Subsequently, univariate Cox proportional hazards regression modeling was performed to identify survival-associated markers. The results showed that 4 potential prognostic genes were significantly associated with OS of GC patients (HR > 1, *P* < 0.001), namely *SERPINE1*, *CD36*, *MMRN1*, and *GRP* (**Figure 2A**). Notably, the quartet of genes successfully met the prerequisites for PH assumption validation, exhibiting *P* > 0.05 (**Supplementary Figure S1**). Consequently, *SERPINE1*, *CD36*, *MMRN1*, and *GRP* were designated as prognostically relevant genetic markers warranting further investigation.

In the RSF model, the 4 genes, including *SERPINE1*, *CD36*, *MMRN1*, and *GRP*, demonstrated high importance (Importance > 0) (**Figure 2B**). In the training set, the 385 GC samples were classified into HRG (n = 192) and LRG (n = 193) based on a median risk score of 42.43821. Notably, the number of deaths among GC patients increased in a progressive manner with increasing risk score (**Figure 2C**). A substantial survival disparity was observed between HRG and LRG via K-M curves, with the HRG demonstrating a marked reduction in survival probability (*P* < 0.0001) (**Figure 2D**). The AUCs for survival at 3, 5, and 7 years were 0.82, 0.85, and 0.80, respectively (**Figure 2E**). The AUC values were all greater than 0.6, and the risk model demonstrated greater predictive power and prognostic value. Similarly, in the validation set, the median risk score of 47.25753 was used to classify 357 GC patients into HRG (n = 178) and LRG (n = 179). Evaluation of the risk score distribution and corresponding survival status (**Figure 2F**), the K-M curves (*P* = 0.0061) (**Figure 2G**), and ROC curve analyses (AUC > 0.6) (**Figure 2H**) confirmed the robustness of the constructed risk model. These findings indicated that the risk model based on *SERPINE1*, *CD36*, *MMRN1*, and *GRP* served as a valuable tool for personalized prognostic assessment in the clinical management of GC.

Analysis of clinical correlations revealed significant variations in risk scores across different N stages, T stages, and overall TNM stages (*P* < 0.05) (**Figure 2I**). However, no significant differences were noted in the other clinical characteristics.

### 3.3 Exploring the functional and immune infiltration differences between HRG and LRG

**Figure 3:**
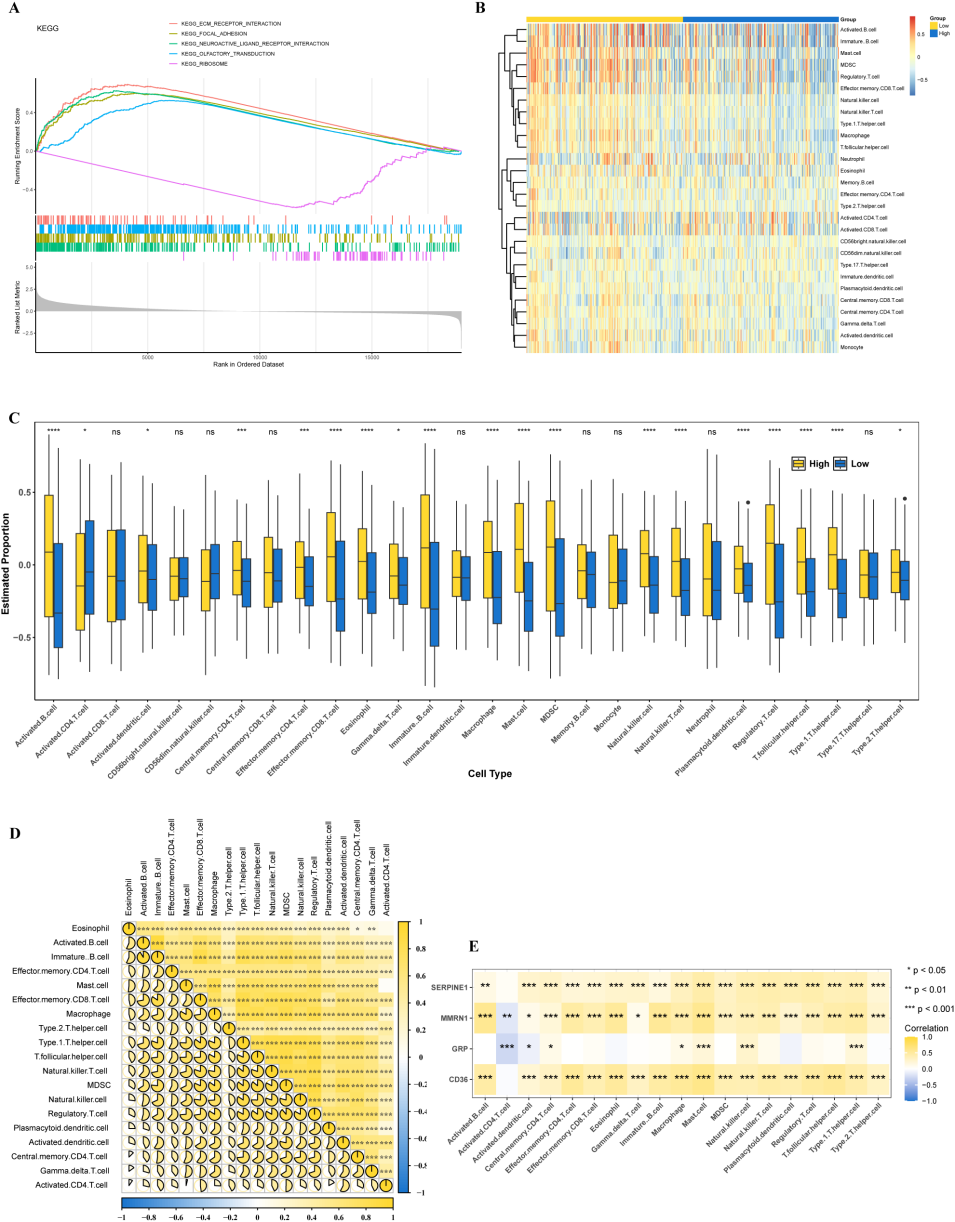
GSEA and immune landscape. **(A)** GSEA. **(B)** Composition of immune cells in the two risk-score subgroups. **(C)** The relative immune infiltration score of 28 immune cells between low- and high-risk groups. **(D)** Correlations among the abundance of immune cells in the proposed model. **(E)** Correlations between the abundance of immune cells and four genes in the proposed model. *P < 0.05, **P < 0.01, ***P < 0.001, ****P < 0.0001.

GSEA revealed distinct transcriptomic signatures between the high- and low-risk cohorts, with 62 biological signaling cascades exhibiting significant differential enrichment patterns, including neuroactive ligand-receptor interaction, ribosome, and ECM receptor interaction (**Figure 3A**, **Supplementary Table S4**). The study results indicated that the most significant pathways were associated with cell signaling pathways, which might be linked to the development of GC.

For HRG and LRG samples, the proportions of 28 immune cell score types are shown in **Figure 3B**. A comparative analysis of the immune cell infiltration score revealed significant disparities among 19 distinct immune cell types between the 2 groups (*P* < 0.05) (**Figure 3C**), such as activated B cells. Most cells exhibiting significant differences demonstrated strong positive correlations with prognostic genes (**Figure 3D**). Among these, the strongest and most significant positive correlations were observed between activated B cells and immature B cells (r > 0.8, *P* < 0.001). Furthermore, CD36, MMRN1, and SERPINE1 demonstrated significant positive correlations with mast cells (r > 0.3, *P* < 0.001) (**Figure 3E**). The aforementioned observations yielded valuable mechanistic understanding regarding the molecular underpinnings driving GC pathogenesis and progression.

### 3.4 Differences in mutations and chemotherapy drug sensitivity between HRG and LRG

**Figure 4:**
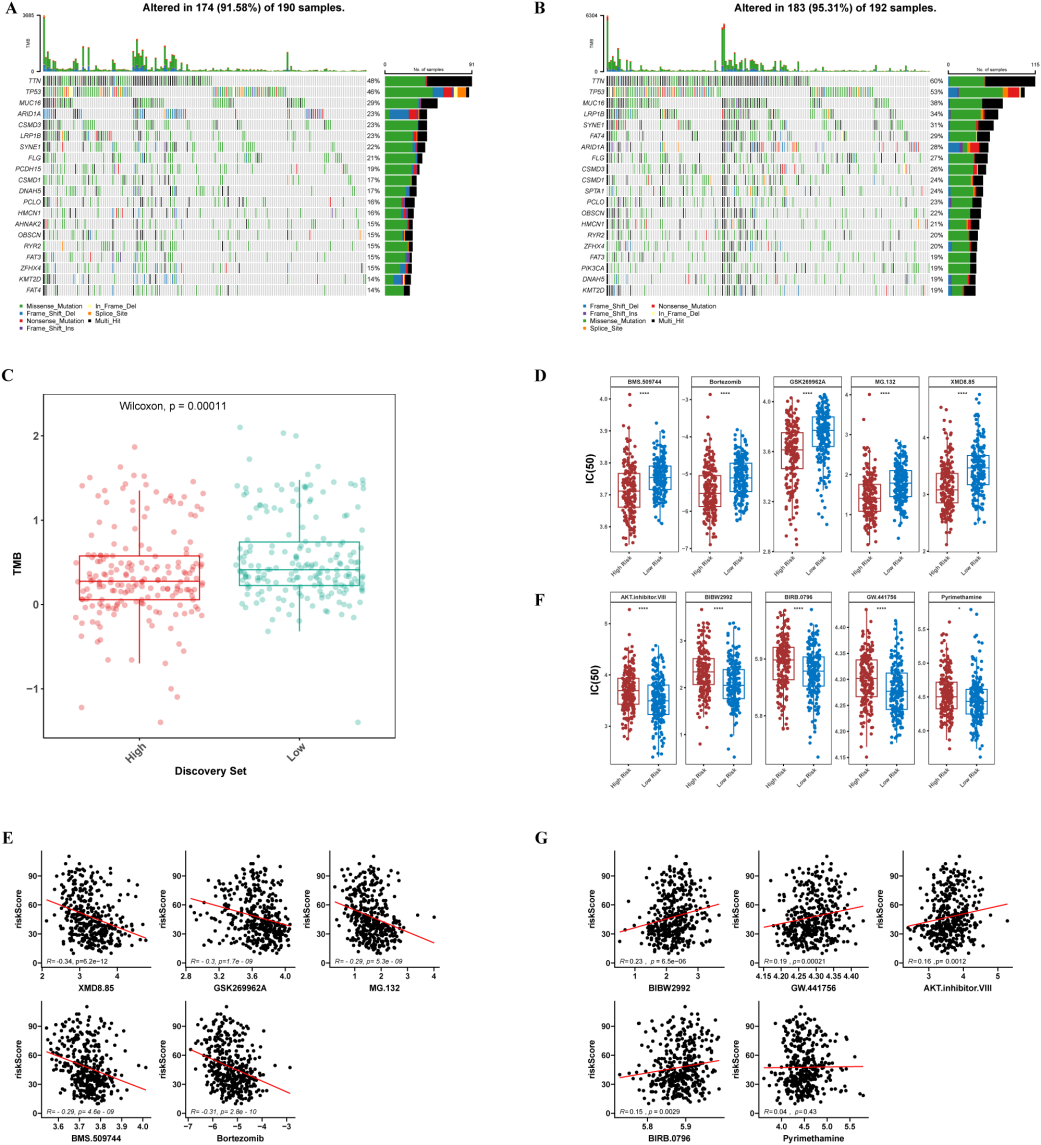
Gene mutation and drug sensitivity. **(A, B)** Significantly mutated genes in the mutated GC samples of the high and the low-risk groups, respectively. **(C)** The TMB of two different risk subgroups. **(D)** Top 5 high expression drugs in low-risk groups. **(E)** Correlation between the top 5 high expression drugs in the low-risk group and the risk score. **(F)** Top 5 high-risk drugs with high expression. **(G)** Correlation between the top 5 high-risk drugs and risk score. *, p<0.05; ****, p<0.0001.

As shown in **Figures 4A, B**, waterfall plots were generated to visualize the mutational landscapes of the HRG and LRG. Within the HRG, the most prevalent mutated genes were *TTN* (48%), *TP53* (46%), *MUC16* (29%), *ARID1A* (23%), and *CSMD* (23%). Conversely, in the LRG, the top 5 mutated genes were *TTN* (60%), *TP53* (53%), *MUC16* (38%), *LRP1B* (34%), and *SYNE1* (31%). Moreover, missense mutation was identified as the predominant mutation type within the two groups. There was a significant difference in GC prognosis between the high- and low-TMB groups (*P* = 0.00011) (**Figure 4C**).

Comparative pharmacological sensitivity profiling revealed that 74 therapeutic agents exhibited significant differences in IC_50_ patterns across the dichotomized risk categories (*P* < 0.05). Notably, 69 of these compounds exhibited substantially lower IC_50_ values in the HRG than in the LRG, suggesting greater therapeutic susceptibility (**Supplementary Table S5**). The top 5 drugs identified were XMD8.85, GSK269962A, MG.132, BMS.509744, and Bortezomib (*P* < 0.0001) (**Figure 4D**).

Besides, correlation analysis demonstrated that three specific agents, XMD8.85, GSK269962A, and Bortezomib, exhibited significant negative associations with the computed risk scores, with correlation coefficients below -0.30 and statistical significance at *P* < 0.05 (**Figure 4E**). Conversely, a subset of five compounds, specifically AKT inhibitor VIII, BIBW2992, BIRB.0796, GW.441756, and Pyrimethamine, exhibited markedly elevated IC_50_ metrics within the HRG when benchmarked against the LRG, indicating reduced pharmacological sensitivity (*P* < 0.05) (**Figure 4F**). Correlation analysis for this quintet of agents revealed no significant associations between IC_50_ measurements and risk stratification scores (**Figure 4G**). Collectively, these pharmacogenomic observations substantiate that the established risk scoring system has clinically meaningful prognostic utility for predicting chemotherapeutic response profiles in GC patient populations, thereby facilitating personalized therapeutic decision-making.

### 3.5 Endothelial cells identified as key cells

**Figure 5:**
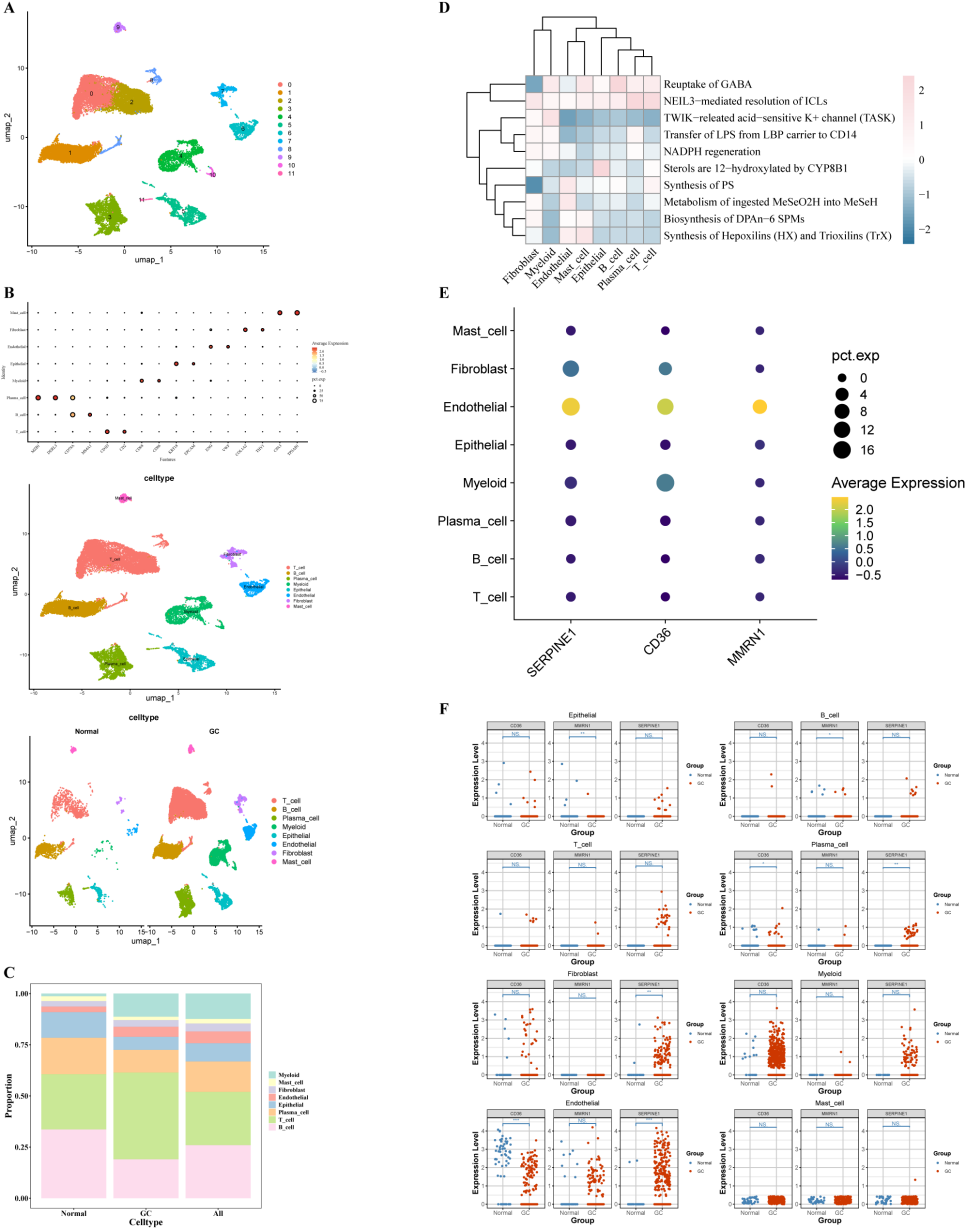
Single-cell RNA-sequencing analysis. **(A)** UMAP plot of 12 cell clusters. **(B)** Scatter plot depicting the distribution of the marker gene for each cluster, and UMAP plot of all clusters with cell-type annotations. **(C)** Cell proportions of subtypes of 8 cells in normal and GC tissues. **(D)** Differential pathway enrichment analysis. **(E)** Bubble plot of prognostic gene expression in cells. **(F)** Differential analysis of prognostic genes in cells. *P < 0.05, **P < 0.01, ***P < 0.001.

For the GSE167297 dataset, pre-filtration cellular composition encompassed 27,073 individual cells alongside 19,887 genetic features. Following implementation of stringent quality assurance criteria, the cellular repertoire was consolidated to 23,177 entries, whereas the gene inventory maintained consistency at 19,887 features (**Supplementary Figure S2A**). Subsequently, 2,000 HVGs exhibiting maximal transcriptional variance were extracted, with the decile of most variable genes encompassing *IGLL5*, *LIPF*, and *PGC* among others (**Supplementary Figure S2B**). Principal component analysis revealed diminishing explanatory power beyond the 30^th^ component, as evidenced by a plateau in the scree plot at PC = 30. Consequently, the initial 30 principal components were retained for subsequent dimensionality reduction procedures (**Supplementary Figure S2C**). Next, cells were partitioned into 12 clusters using UMAP (**Figure 5A**). Based on the expression of marker genes, the 12 cell clusters were annotated into 8 cell types: myeloid cells, endothelial cells, T cells, B cells, fibroblasts, plasma cells, epithelial cells, and mast cells (**Figure 5B**). In all samples, T cells and B cells comprised the predominant cellular populations (**Figure 5C**). In the normal group, B cells represented a larger proportion than T cells; conversely, in the GC group, T cells constituted a significantly greater proportion than B cells. The functional enrichment results showed that the cells were markedly enriched in the substance synthesis and metabolism pathways (*P* < 0.05) (**Figure 5D**, **Supplementary Table S6**). Of these, endothelial cells, mast cells, and T cells were significantly enriched in the pathways for the “TWIK-related acid-sensitive K+ channel (TASK)”. In contrast, fibroblasts showed significant enrichment in the pathways for the “Synthesis of PS” and “Reuptake of GABA”.

The expression levels of SERPINE1, CD36, and MMRN1 were analyzed in 8 cell types (GRP was not expressed in the GSE167297 set) (**Figures 5E, F**). Prognostic genes (*SERPINE1*, *CD36*, and *MMRN1*) were highly expressed in endothelial cells, with 2 of these genes (*SERPINE1* and *CD36*) exhibiting significant differential expression (*P* < 0.05). Endothelial cells, together with other stromal cells, reportedly play a significant role in the initiation and progression of GC. Therefore, endothelial cells were identified as key cells in this context.

**Figure 6:**
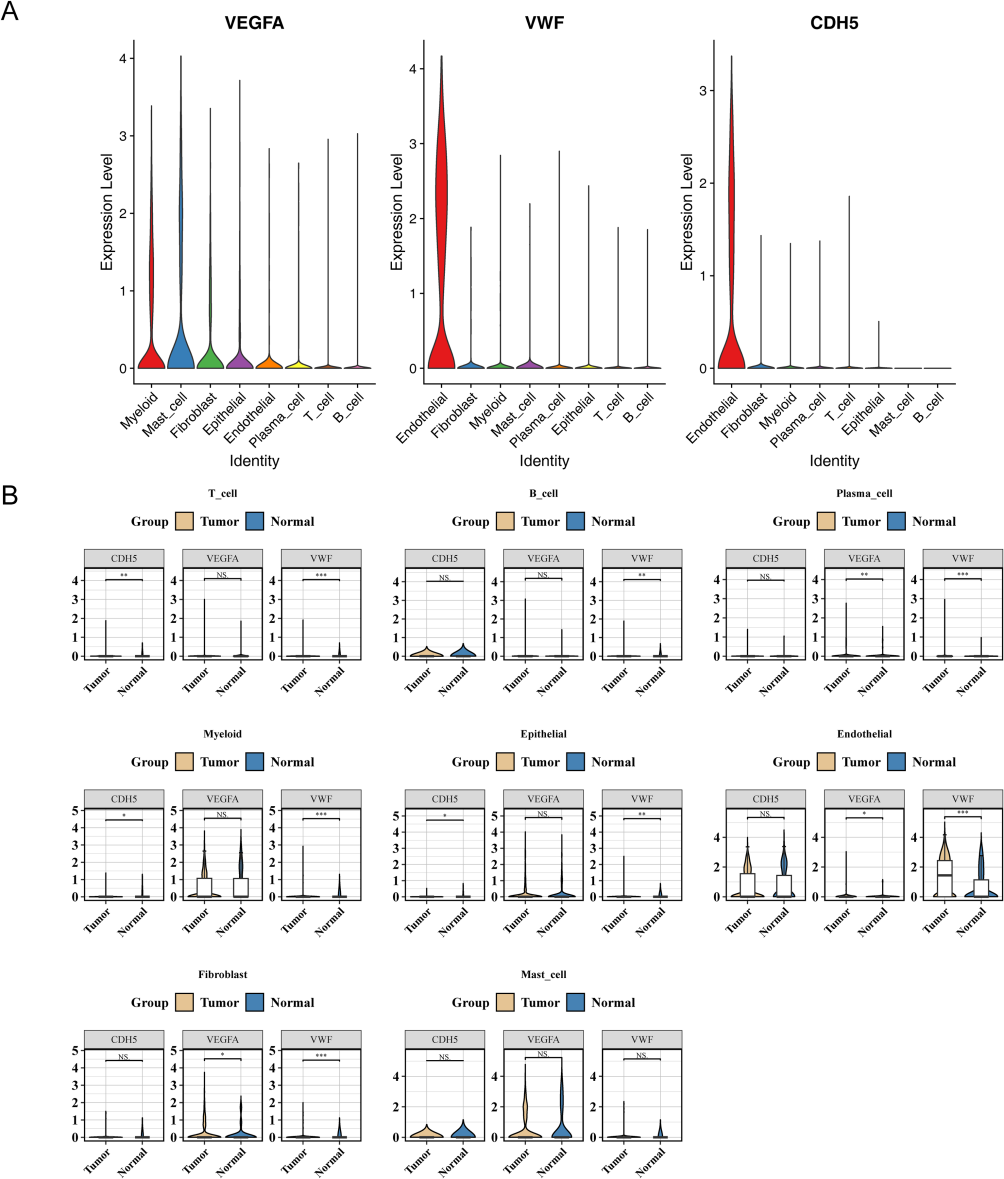
Analysis of endothelial cell marker expression at the single-cell level. **(A)** Violin plot showing the expression of endothelial cell markers (VEGFA, VWF, CDH5) in different cell types. **(B)** Box plot showing the expression differences of endothelial cell markers (VEGFA, VWF, CDH5) between the disease group and the control group. Ns stands for not significant, *P < 0.05, **P < 0.01, ***P < 0.001.

To clarify the expression patterns of endothelial cells in the gastric cancer microenvironment, we analyzed the expression profiles of classical endothelial cell markers, including VEGFA, VWF and CDH5. The analysis results showed that VWF and CDH5 exhibited high, specific expression in endothelial cells, with significant differences compared with other cell types (**Figure 6A**). In addition, when comparing gastric cancer tissues with normal tissues, we found that the expression levels of VEGFA and VWF differed significantly between the two groups (**Figure 6B**). These findings suggested that these molecules might be involved in gastric cancer-associated angiogenesis or microenvironmental remodeling, thereby providing evidence for the critical role of endothelial cells in gastric cancer progression.

### 3.6 Exploring the interactions, differentiation, and CNVs of endothelial cells

**Figure 7:**
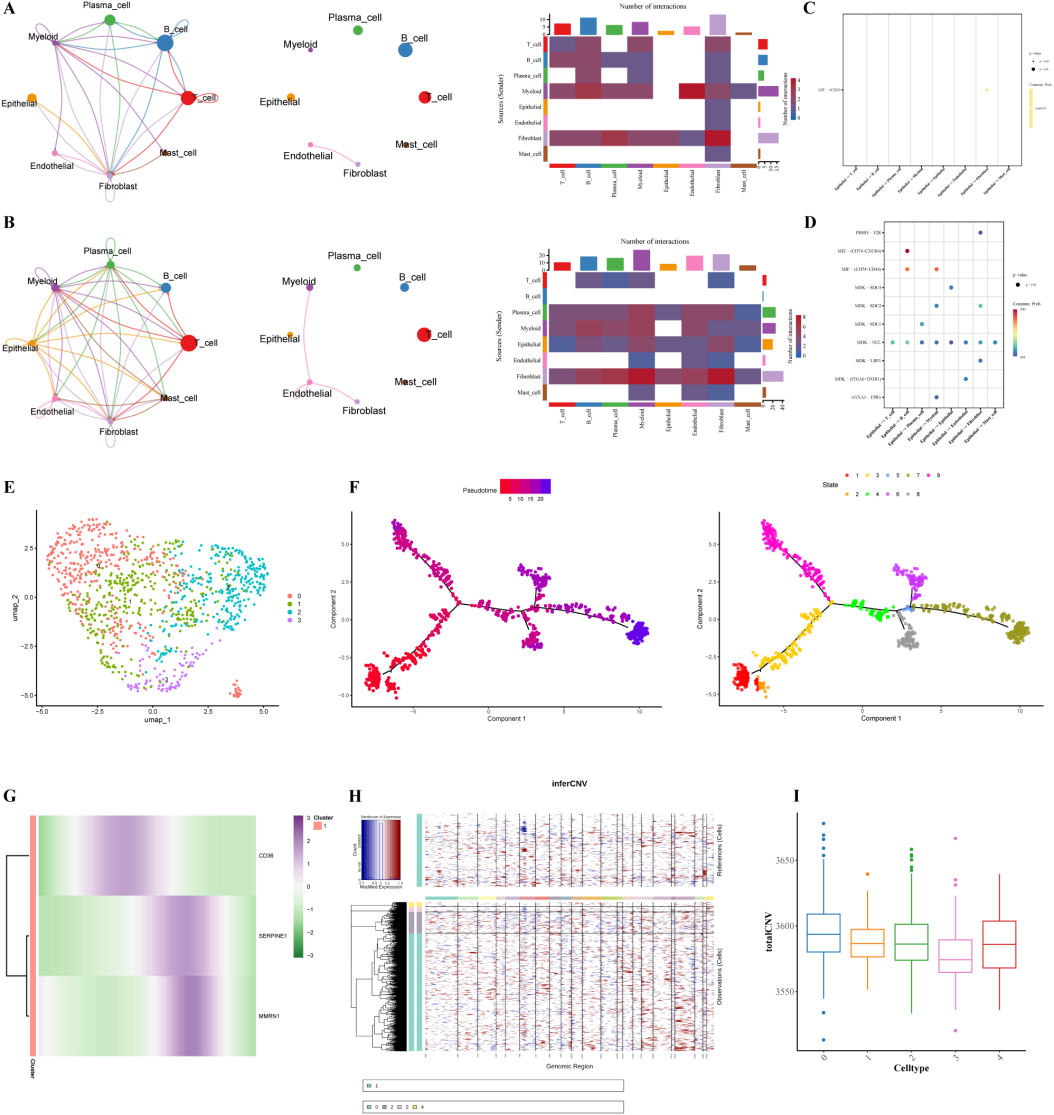
Cell communication, cell developmental trajectory, and malignant analyses. **(A)** Normal histiocytocyte ligand and receptor interaction network diagram (analyzed using the GSE167297 single-cell dataset). **(B)** GC cell ligand-receptor interaction network. **(C)** Analysis of normal cell-to-cell ligand-receptor communication probability. **(D)** Analysis of communication probability between ligand and receptor in GC cells. **(E)** Endothelial cell clustering diagram. **(F)** Endothelial pseudo-periodic analysis. **(G)** Expression trend of prognostic gene in Endothelial Cells. **(H)** InferCNV result heat map. **(I)** Cluster before annotation of cell type.

Comprehensive interrogation of intercellular communication networks demonstrated that the eight characterized cellular subpopulations within glioma specimens exhibited both elevated frequency and amplified intensity of signaling interactions relative to their normal tissue counterparts. Within the normal group, the endothelial cells emitted few signals but received numerous low-strength signals, maintaining connections exclusively with fibroblasts (**Figure 7A**). In the GC sample, endothelial cells emitted few signals but received relatively more signals with lower connection strengths, maintaining connections with themselves as well as fibroblasts and myeloid cells (**Figure 7B**). Furthermore, the MIF-ACKR3 signaling axis was identified as a key mediator in the interactions between epithelial cells and fibroblasts in the normal group, while the MIF-(CD47 +CXCR4) signaling axis was crucial for interaction between epithelial cells and B cells in the GC group (**Figures 7C, D**). These findings suggested distinct signaling mechanisms underlying cell communication in different disease contexts.

Further analysis of endothelial cells through secondary dimensionality reduction and clustering revealed 4 distinct subgroups (**Figure 7E**). Pseudotime analysis of endothelial cell differentiation uncovered a progressive trajectory from an early (dark purple) to a more mature (light red) state, and the 4 subgroups could be roughly divided into 9 differentiation states (**Figure 7F**). During endothelial cell differentiation, CD36 showed elevated expression in the early-to-mid stages, with minimal expression in other phases. SERPINE1 and MMRN1 exhibited peak expression from the mid to late stages, remaining low during other developmental periods (**Figure 7G**). These findings collectively suggested the potential involvement of SERPINE1, CD36, and MMRN1 in different phases of endothelial cell differentiation in GC.

During the analysis of malignant cells, cluster 1 was designated as the reference cell type (mast cells), while clusters 0, 2, 3, and 4 were initially unannotated as endothelial cells. Compared with the reference cell cluster (cluster 1), no significant CNVs were observed in clusters 0, 2, 3, or 4 (**Figures 7H, I**). This finding indicates the absence of malignant cells within the endothelial cell population.

### 3.7 The function and regulatory relationships of prognostic genes

**Figure 8:**
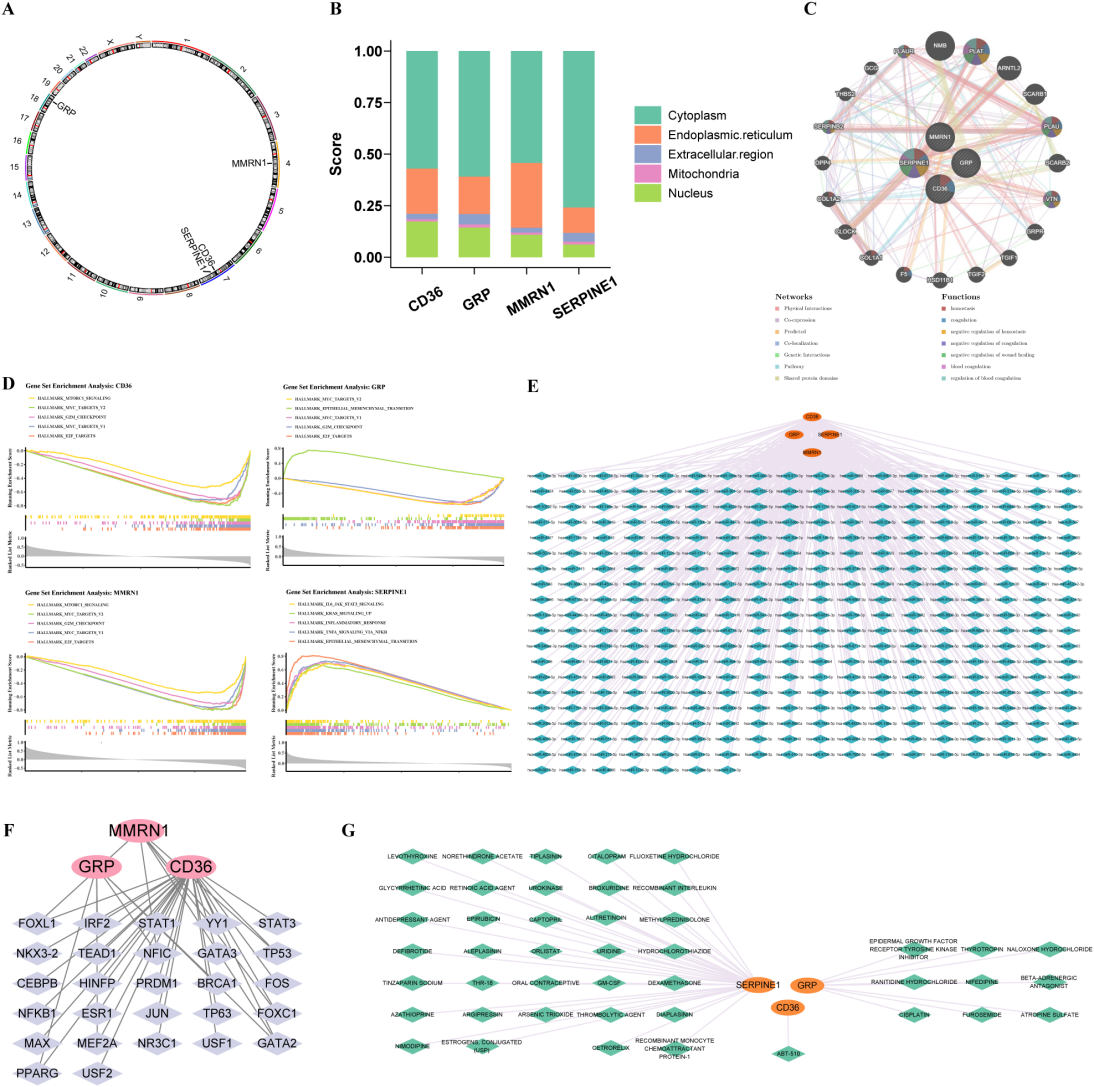
The function and regulatory relationships of prognostic genes. **(A)** The location of prognostic genes on chromosomes. **(B)** Subcellular localization of prognostic genes. **(C)** GeneMania-based interaction network for prognostic genes. **(D)** Single gene GSEA analysis. **(E)** miRNA-mRNA network. **(F)** TF-mRNA network. **(G)** Prognostic genes and drug network.

Chromosomal localization results indicated that *SERPINE1* and *CD36* were located on chromosome 7, *MMRN1* on chromosome 4, and *GRP* on chromosome 18 (**Figure 8A**). Moreover, all prognostic genes were primarily located in the cytoplasm (**Figure 8B**). The GeneMANIA network revealed that the prognostic genes interacted with 20 others (PLAT, PLAU, VTN) and were collectively enriched for functions related to coagulation, including hemostasis and its negative regulation (anticoagulation) (**Figure 8C**). Subsequent GSEA identified 35, 28, 30, and 26 pathways significantly enriched in correlation with the expression levels of CD36, GRP, MMRN1, and SERPINE1, respectively (P<0.05) (**Supplementary Table S7**). Notably, CD36, GRP, and MMRN1 were significantly enriched in E2F targets, MYC targets V1, G2M checkpoint, and MYC targets V2, while SERPINE1 was significantly enriched in epithelial-mesenchymal transition, TNFA signaling via NFKB, KRAS signaling up, inflammatory response, and IL6 JAK STAT3 signaling (**Figure 8D**). These results suggested that prognostic genes were associated with significant alterations in cell signaling pathways and immune responses, which might correlate with the characteristics of GC.

Computational prediction of miRNA–mRNA regulatory networks revealed distinct post-transcriptional landscapes: SERPINE1 was identified as a putative target of 118 miRNAs, while CD36, MMRN1, and GRP were predicted to be regulated by 152, 88, and 30 miRNA species, respectively (**Figure 8E**). Notably, hsa-miR-4753-3p emerged as a convergent regulator orchestrating the co-expression of CD36, GRP, and SERPINE1. Transcriptional regulatory network reconstruction identified 25 TFs potentially governing CD36 expression, while MMRN1 and GRP each appeared subject to transcriptional control by 6 distinct TFs, respectively (**Figure 8F**). GATA2 was identified as a co-regulator of MMRN1, GRP, and CD36. This co-targeting indicated that these TFs and miRNAs might have coordinated roles in regulating CD36, GRP, MMRN1, and SERPINE1 expression, highlighting their potential importance in the regulatory network underlying disease pathogenesis. Potential drug targets of prognostic genes for GC were obtained from the DGIdb database, as shown in **Figure 8G**. These included 34 drugs (e.g., DIAPLASININ, LEVOTHYROXINE) that targeted SERPINE1, 1 drug (ABT-510) that targeted CD36, and 9 drugs (e.g., CISPLATIN, ATROPINE SULFATE) that targeted GRP. These findings suggest that the identified compounds may exert therapeutic effects in GC by modulating the expression or activity of key prognostic genes.

### 3.8 SERPINE1 expression correlated with GC prognosis

**Figure 9:**
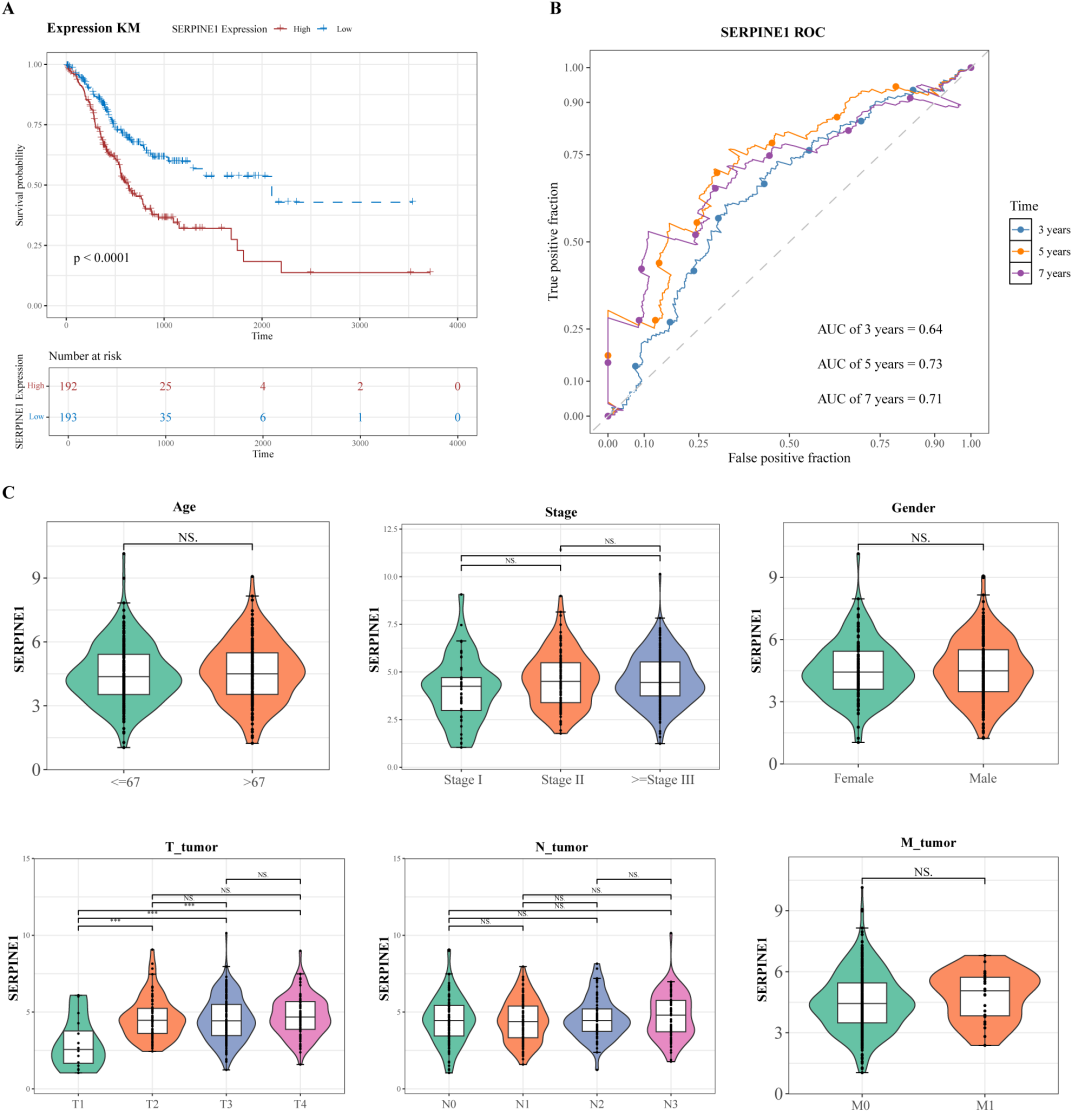
The expression of SERPINE1 was correlated with the prognosis of GC. **(A)** K-M survival curve of SERPINE1. **(B)** SERPINE1 ROC analysis curve at 3, 5, and 7 years. **(C)** The differences in risk scores among different clinical characteristic groups. The different colors in the figure represent the subtypes of the same clinical characteristic. The name of the clinical characteristic appears above each box plot. The horizontal axis shows the different subtypes of the clinical characteristic, and the vertical axis shows the risk score (riskScore).

In the training set, the 385 GC samples were classified into HRG (n = 192) and LRG (n = 193) according to the median SERPINE1 expression of 4.450664. In the prognostic analysis, SERPINE1 overexpression was significantly associated with a poorer prognosis in GC (*P* < 0.0001) (**Figure 9A**). Furthermore, the AUC values of the ROC curves for the 3-, 5-, and 7-year survival rates in the training set all exceeded 0.6, indicating that the *SERPINE1* gene exhibited favorable prognostic predictive performance (**Figure 9B**). Based on the median SERPINE1 expression of 4.450664, the 385 GC samples were classified into HRG (n = 172) and LRG (n = 176). SERPINE1 expression levels showed significant differences between Stage I and ≥ Stage III, as well as between T1 and T2, T1 and T3, and T1 and T4 (*P* < 0.05) (**Figure 9C**). Furthermore, elevated SERPINE1 expression was associated with T stage (*P* = 0.039) (**Table 1**). Therefore, SERPINE1 expression was associated with clinical features and prognosis in GC.

**Table 1:** Baseline clinical characteristics by SERPINE1 expression group.

Clinicopathologic characteristic	SERPINE1			*P*-value
	High (172)		Low (176)	
T_tumor (%)	T1	3(1.7)	13(7.4)	0.039
	T2	36(20.9)	35(19.9)	
	T3	78(45.3)	86(48.9)	
	T4	55(32.0)	42(23.9)	
N_tumor (%)	N0	55(32.0)	55(31.2)	0.803
	N1	43(25.0)	51(29.0)	
	N2	35(20.3)	36(20.5)	
	N3	39(22.7)	34(19.3)	
M_tumor (%)	M0	158(91.9)	167(94.9)	0.357
	M1	14(8.1)	9(5.1)	
Stage (%)	Stage I	18(10.5)	28(15.9)	0.277
	Stage II	60(34.9)	53(30.1)	
	≥ Stage III	94(54.7)	95(54.0)	
Gender (%)	Female	59(34.3)	67(38.1)	0.536
	Male	113(65.7)	109(61.9)	
Age (%)	≤ 67	87(50.6)	100(56.8)	0.29
	> 67	85(49.4)	76(43.2)	

### 3.9 qRT-PCR validation of prognostic genes

**Figure 10:**
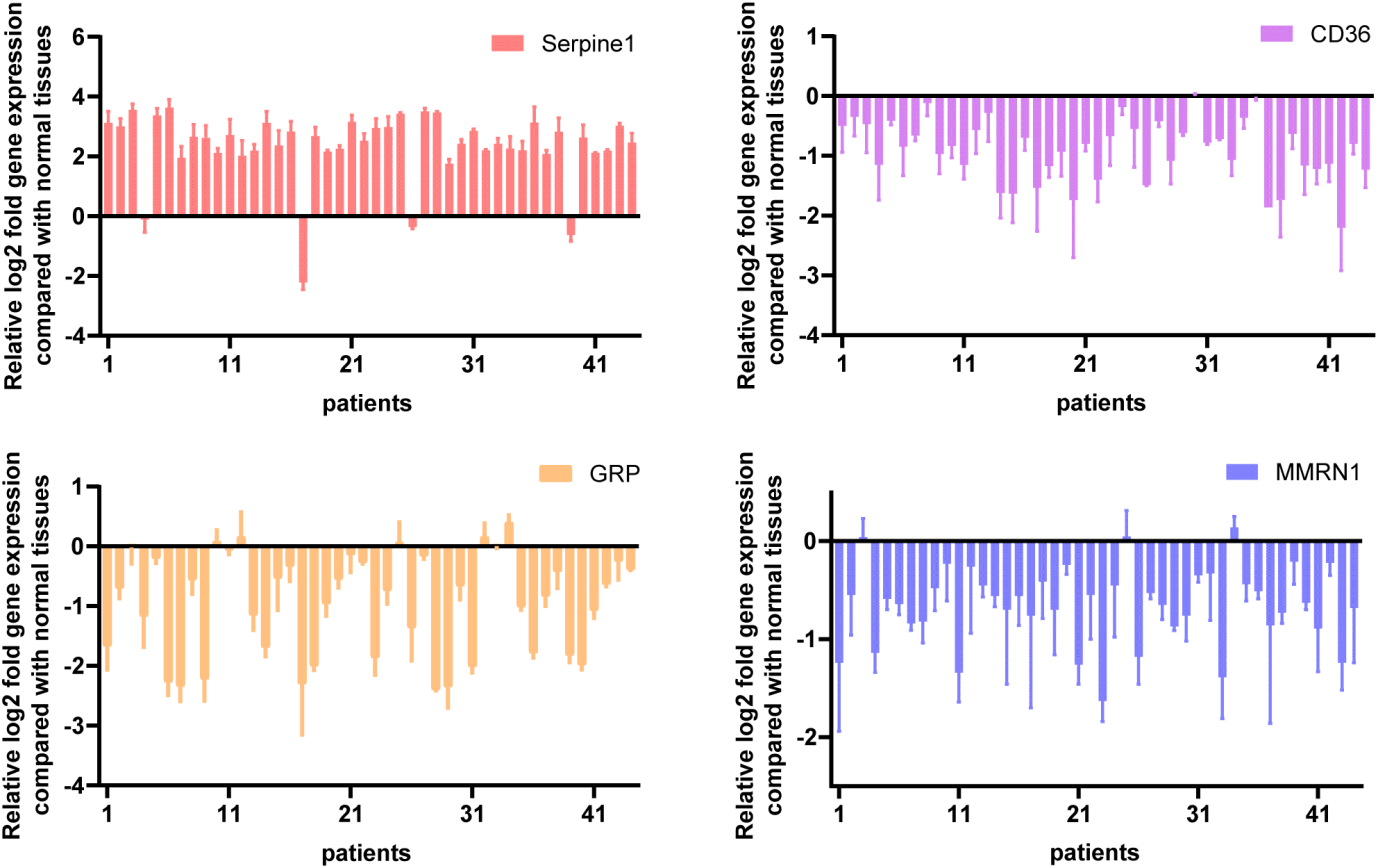
qRT-PCR quantification of mRNA expression levels of prognostic genes in GC. mRNA levels of four potential prognostic genes were quantified by qRT-PCR in 44 paired GC and adjacent normal tissue samples.

Experimental corroboration of expression profiles for the quartet of candidate prognostic markers was performed using qRT-PCR on 44 matched pairs of primary GC specimens and their corresponding adjacent non-malignant gastric tissues. Quantitative assessment demonstrated divergent transcript abundance patterns between malignant and benign tissue compartments (**Figure 10**). *SERPINE1* was upregulated in 40 of the 44 GC samples (90.9%), while *CD36* was consistently downregulated across all 44 samples (100%). *MMRN1* was downregulated in 41 of the 44 samples (93.2%), and *GRP* was downregulated in 39 of the 44 samples (88.6%). These findings collectively demonstrated the differential expression of SG-associated prognostic genes in GC tissues. Notably, *SERPINE1* was predominantly upregulated, whereas *CD36*, *MMRN1*, and *GRP* were mainly downregulated compared to adjacent normal tissues.

## 4 Discussion

GC continues to impose a substantial burden on global healthcare systems, currently positioned as the world’s fifth most frequently diagnosed malignancy while simultaneously representing the fourth leading contributor to oncology-associated fatalities worldwide (2). Despite rapid advances in therapeutic modalities, the prognosis for advanced GC remains dismal (42). Therefore, understanding the molecular mechanisms underlying GC tumorigenesis is critical for improving patient outcomes. SGs represent dynamic ribonucleoprotein complexes that form under stress conditions and can be induced by chemotherapeutic agents (43, 44). GC cells can form SGs when relevant pathways are activated to reduce cell apoptosis (17). The mechanism of action of SGs in tumors has become a research focus, but the exact mechanism by which SGs contribute to the pathogenesis of GC remains unclear. In this comprehensive study, we identified 124 stress granule-related genes differentially expressed between GC and normal tissues, establishing a four-gene prognostic signature with robust predictive accuracy validated across independent datasets.

Among the four identified prognostic genes, *SERPINE1* serves as the primary inhibitor of urokinase and tissue plasminogen activators, maintaining hemostatic balance (45). High-throughput sequencing has revealed aberrant *SERPINE1* expression across multiple malignancies, including GC, where it functions as an oncogenic driver by enhancing cellular proliferation, migration, invasion, and angiogenesis (46, 47). Recent studies have demonstrated significant *SERPINE1* overexpression in GC with considerable prognostic potential (48, 49), consistent with the RT-qPCR results of this study. Compared to control samples, the expression of SERPINE1 was significantly upregulated in GC samples. Notably, recent evidence has established a direct mechanistic relationship between SERPINE1 and stress granules, wherein SGs sequester PAI-1 (SERPINE1) to modulate cellular senescence and stress responses (50). This mechanistic finding provides a molecular foundation for understanding how *SERPINE1* influences GC progression through stress granule-mediated pathways.

CD36 is now understood to function as a scavenger receptor that facilitates fatty acid uptake and participates in metabolic reprogramming, enabling cancer cells to adapt to nutrient-depleted tumor microenvironments while promoting invasion and metastasis (51, 52). MMRN1 represents a platelet and endothelial cell protein involved in hemostasis that also regulates tumor angiogenesis by modulating endothelial cell function and vascular remodeling processes (53, 54). Gastrin-releasing peptide (GRP) acts as a neuropeptide hormone with established roles in gastrointestinal physiology and significant functions in promoting cancer cell proliferation, invasion, and resistance to apoptosis (55).

Using these four prognostic genes, our predictive model demonstrated robust performance across training and validation cohorts. Kaplan-Meier survival analysis confirmed significantly poorer outcomes in high-risk patients, while ROC analysis yielded AUC values exceeding 0.6 across multiple time points, indicating strong prognostic capability. These findings aligned with established prognostic models in GC, including lactylation scores for predicting immune escape and cancer-associated fibroblast signatures for immune microenvironment assessment (56, 57). Our four-gene prognostic signature provides a reliable prognostic biomarker that could facilitate early risk stratification and personalized therapeutic decision-making in clinical practice.

GSEA revealed significant pathway differences between risk groups, notably epithelial-mesenchymal transition (EMT), extracellular matrix-receptor interaction, and TNF-α signaling via NF-κB. EMT is a critical process whereby epithelial cells lose polarity and gain mesenchymal characteristics, enhanced by signals from the tumor microenvironment (58). In GC, tumor-associated fibroblasts secrete factors that activate TGF-β signaling, inducing EMT and enhancing invasive capabilities (59, 60). Our results suggest that multiple genes in our prognostic signature may work synergistically to regulate EMT. For instance, SERPINE1 may participate in TGF-β pathway regulation, where upregulated expression enhances downstream transcription factor activation, ultimately promoting EMT and GC cell invasiveness.

Immune infiltration analysis identified significant differences in 19 immune cell types between risk groups, with significant positive correlations among CD36, MMRN1, SERPINE1, and mast cell infiltration levels. Mast cells function as tissue-resident immune sentinel cells, secreting cytokines such as TNF-α and IL-8 that can either enhance anti-tumor immunity or promote tumor-favorable microenvironments (61, 62). In GC, mast cell-derived factors may recruit additional immune cells while potentially facilitating immune escape mechanisms (63). Furthermore, our analysis revealed significant enrichment differences in activated B cells, dendritic cells, and regulatory T cells, suggesting complex immune microenvironmental remodeling associated with our prognostic signature. This multi-cellular immune landscape indicates that prognostic genes may synergistically influence tumor immunity through diverse cellular interactions, simultaneously affecting both anti-tumor responses and immune tolerance.

In the present study, drug-sensitivity analysis revealed differential therapeutic responses across risk groups, providing valuable insights for precision medicine. High-risk patients demonstrated enhanced sensitivity to compounds including bortezomib, GSK269962A, and XMD8.85, while low-risk patients showed preferential responses to AKT inhibitor VIII, BIBW2992, and BIRB.0796. The enhanced sensitivity of high-risk patients to proteasome inhibitors such as bortezomib may be associated with the distinct molecular characteristics identified in our study. Firstly, Bortezomib, an FDA-approved proteasome inhibitor, induces G2/M arrest and apoptosis through NF-κB inhibition and induction of endoplasmic reticulum stress (64). High-risk tumors that rely on this signaling axis are thus more vulnerable to disruption. Our GSEA results (**Figure 8D**) revealed that the high-risk signature, particularly SERPINE1, was significantly enriched in ‘TNFA signaling via NFKB’. Secondly, proteasome inhibition itself can trigger stress granule formation as a cellular adaptive response to proteotoxic stress (65). Stress granule-related genes may modulate cellular sensitivity to Bortezomib by regulating intracellular stress responses and protein degradation pathways, which are critical processes in maintaining cellular homeostasis and adapting to therapeutic stress. These differential drug sensitivities are based on *in silico* pharmacogenomic predictions from the GDSC database and serve as experimental hypotheses for potential therapeutic strategies that require subsequent preclinical and clinical validation. Indeed, they should not be used directly as clinical treatment recommendations.

In the present study, single-cell analysis identified endothelial cells as key cellular drivers of GC progression based on prognostic gene expression patterns. Endothelial cells reportedly facilitate tumor metastasis by promoting cancer cell intravasation and by serving as sources of cancer-associated fibroblasts, thereby promoting invasive behaviors (66, 67). In GC, gastric cancer mesenchymal stem cells stimulate endothelial cell proliferation and angiogenesis while promoting tumor cell migration through Slit2 expression (68). Our results identified high SERPINE1 expression in endothelial cells, which is particularly significant given SERPINE1’s enrichment in EMT pathways. This suggests that during GC progression, elevated SERPINE1 expression may initiate EMT processes while promoting angiogenic factor secretion, creating positive feedback loops that enhance both tumor growth and metastatic potential.

In conclusion, this study successfully identified four stress granule-related prognostic genes (*SERPINE1*, *CD36*, *MMRN1*, and *GRP*) that were significantly associated with gastric cancer development and progression, particularly in regulating tumor proliferation and metastasis. The prognostic model constructed from these genes exhibits exceptional predictive performance, achieving AUCs of 0.82, 0.85, and 0.80, for overall survival at 3, 5, and 7 years respectively, in the training cohort, with superior accuracy at 5 years compared to previously reported gastric cancer prognostic models (69). This performance advantage, validated across independent cohorts, underscores the model’s robust utility in patient stratification and its potential to guide therapeutic decision-making. Beyond predictive performance, the model introduces mechanistic novelty by being the first to focus on stress granules, an emerging biological process closely associated with tumor stress adaptation, thereby overcoming the conventional limitations in research perspectives observed in existing models. From a clinical perspective, the high- and low-risk stratification defined by the model not only effectively distinguishes prognostic differences among patients but also identifies high-risk patients as more sensitive to chemotherapy drugs such as Bortezomib. Furthermore, single-cell analysis identified endothelial cells as key drivers of gastric cancer progression, offering an integrated framework encompassing prognostic prediction, drug selection, and target localization for the development of individualized treatment strategies. In contrast, existing comparative models primarily emphasize prognostic evaluation or describe immune infiltration characteristics (56, 57) without providing direct therapeutic guidance. In summary, the proposed model achieves synergistic integration of predictive power, mechanistic innovation, and clinical translational utility, establishing its role as a robust tool for advancing precision medicine in gastric cancer.

Nevertheless, several important limitations warrant acknowledgment. First, the precise mechanistic pathways through which the identified prognostic markers exert their biological effects require rigorous validation in both cell-based experimental systems and animal models to establish causative relationships. Secondly, the robustness of the prognostic model needs to be validated in prospective, multi-center, and large-scale populations. Besides, our study relied on retrospective data from public databases, which may introduce selection bias, and the limited sample size in single-cell analysis potentially affects the generalizability of cellular subpopulation findings. Finally, gene-drug association analyses were predominantly based on computational predictions, and their therapeutic potential requires experimental and clinical validation.

Future investigations should focus on functional experiments to elucidate SG-mediated progression, prospective multi-center validation of our prognostic model, and the development of targeted therapies. Furthermore, integrating multi-omics data will be essential to resolve the regulatory complexities of SGs in the tumor microenvironment and advance personalized oncology.

## 5 Conclusion

This study identified four stress granule-related gastric cancer prognostic genes (*SERPINE1*, *CD36*, *MMRN1*, and *GRP*). The established gene-based prognostic model exhibited reliable performance in predicting survival outcomes for GC patients. Besides, single-cell transcriptomic profiling revealed the essential role of endothelial cells in GC progression. These results offer valuable insights into the molecular basis of GC pathogenesis and may guide the development of novel targeted therapeutic strategies.

## Data Availability

The datasets presented in this study can be found in online repositories. The names of the repository/repositories and accession number(s) can be found in the article/**Supplementary Material.**

## Supplementary material

Supplementary Figure 1 PH hypothesis test residual plot.

Supplementary Figure 2 Single-cell data quality control. **(A)** Quality control. **(B)** High variability gene screening. **(C)** PCA dimension reduction.

Supplementary Table 1 Stress granules-related genes.

Supplementary Table 2 GO terms for the candidate genes.

Supplementary Table 3 KEGG pathways for the candidate genes.

Supplementary Table 4 GSEA enrichment pathways.

Supplementary Table 5 The IC50 values of 69 drugs in the high-risk group were significantly lower than those in the low-risk group.

Supplementary Table 6 Cell enrichment pathways.

Supplementary Table 7 GSEA enrichment pathways of prognostic genes.

Supplementary Table 8 The primers used for qRT-PCR.
