# Supplementary figures and images for: Unveiling prognostic genes and regulatory mechanisms of stress granules in gastric cancers: an integrated analysis of bulk transcriptomics and single-cell RNA sequencing
Source: Front Oncol. 2026 Feb 18;16:1750088. doi: 10.3389/fonc.2026.1750088 (PMC12956643; doi:10.3389/fonc.2026.1750088)

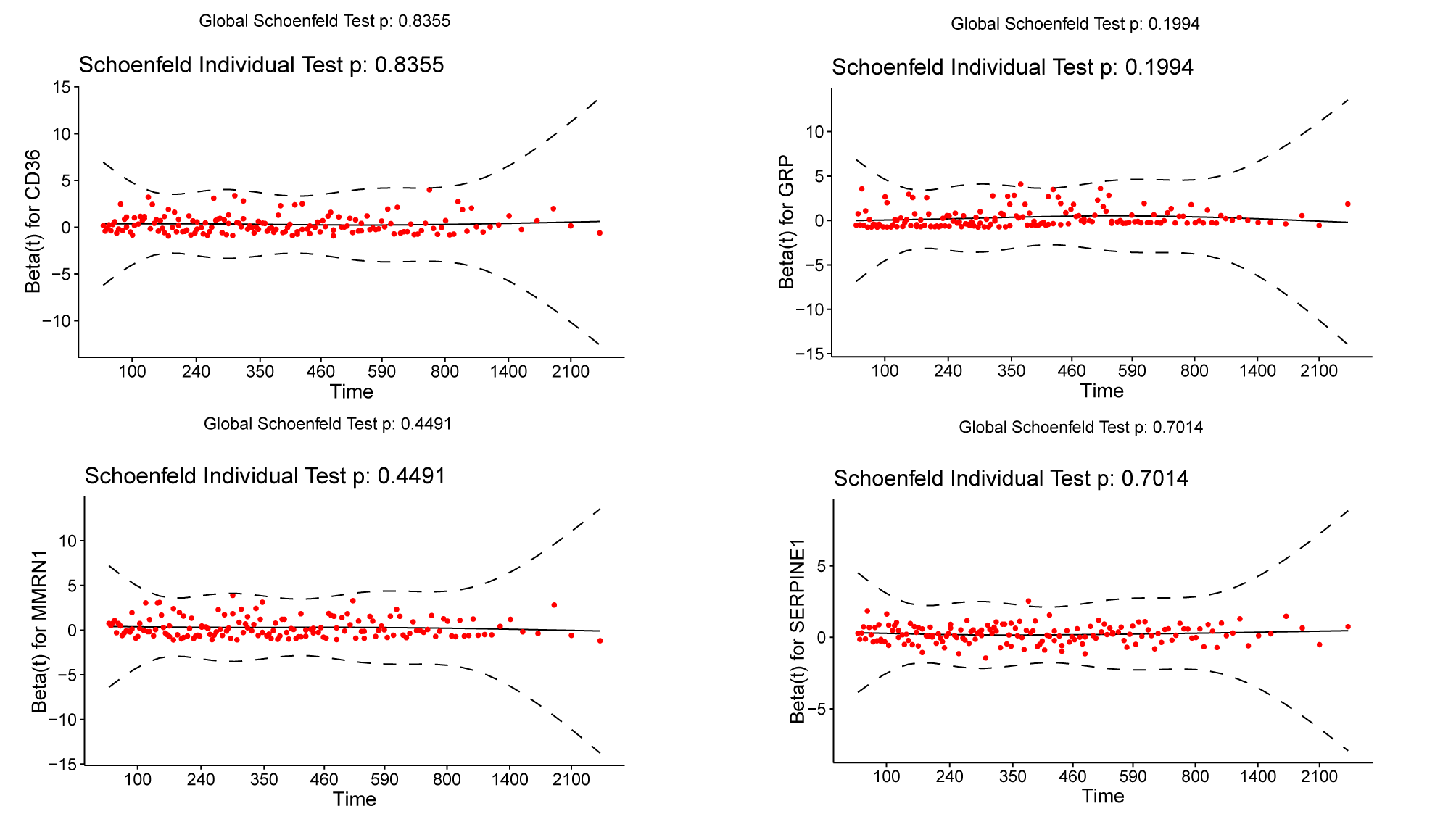

Supplement: Supplementary Figure 1 — PH hypothesis test residual plot. [file Image1.tif]

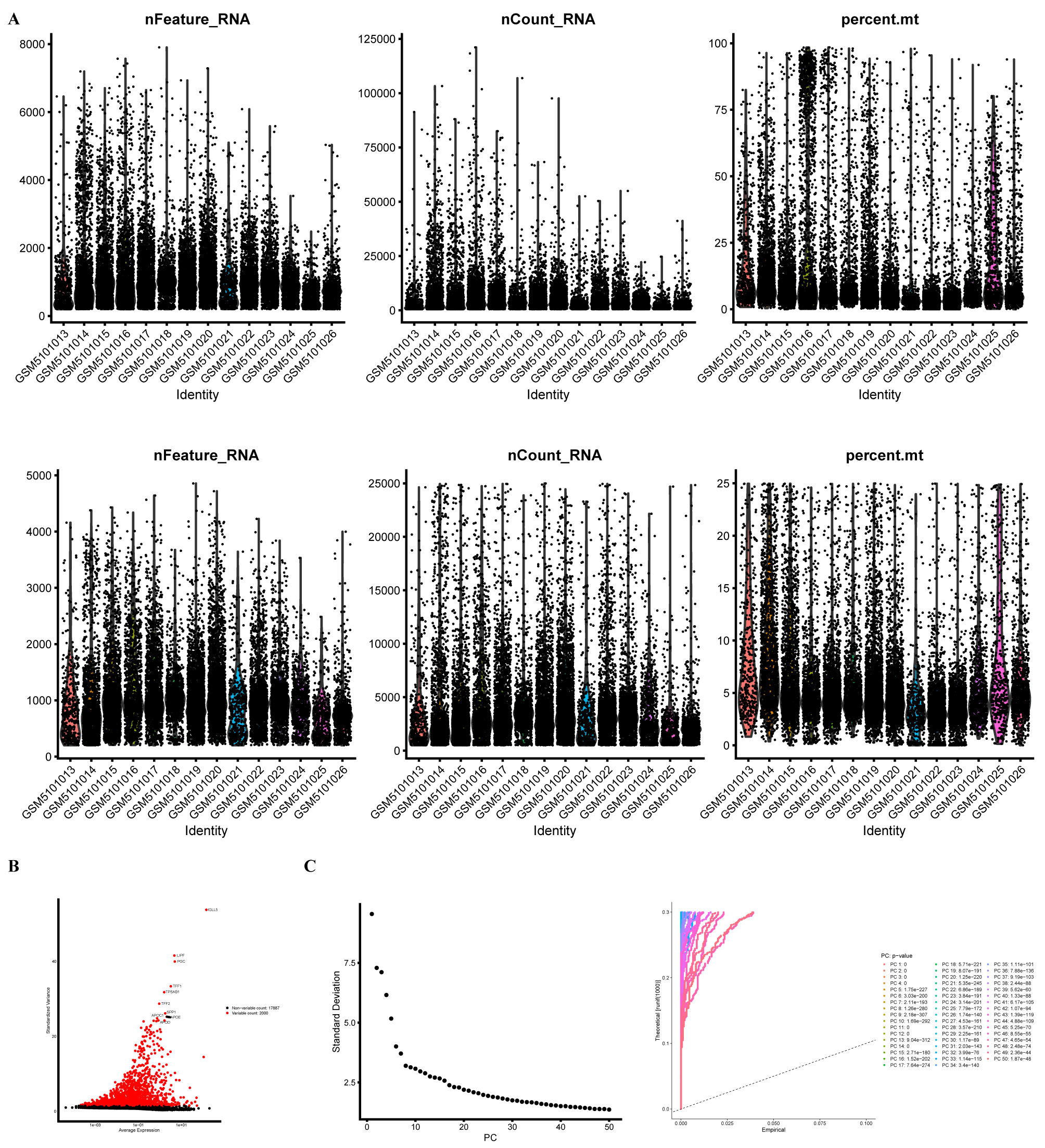

Supplement: Supplementary Figure 2 — Single-cell data quality control. (A) Quality control. (B) High variability gene screening. (C) PCA dimension reduction. [file Image2.tif]
